# Persister Escherichia coli Cells Have a Lower Intracellular pH than Susceptible Cells but Maintain Their pH in Response to Antibiotic Treatment

**DOI:** 10.1128/mBio.00909-21

**Published:** 2021-07-20

**Authors:** Olivia Goode, Ashley Smith, Ashraf Zarkan, Jehangir Cama, Brandon M. Invergo, Daaniyah Belgami, Santiago Caño-Muñiz, Jeremy Metz, Paul O’Neill, Aaron Jeffries, Isobel H. Norville, Jonathan David, David Summers, Stefano Pagliara

**Affiliations:** a Living Systems Institute, University of Exeter, Exeter, United Kingdom; b School of Biosciences, College of Life and Environmental Sciences, University of Exeter, Exeter, United Kingdom; c Department of Genetics, University of Cambridge, Cambridge, United Kingdom; d College of Engineering, Mathematics and Physical Sciences, University of Exeter, Exeter, United Kingdom; e Translational Research Exchange at Exeter, University of Exeter, Exeter, United Kingdom; f MRC Laboratory of Molecular Biology, Cambridge, United Kingdom; g Dstl, Porton Down, Salisbury, United Kingdom; Racah Institute of Physics and the Harvey, The Hebrew University of Jerusalem, Israel; Emory University

**Keywords:** indole, intracellular pH, microfluidics, single-cell analysis, antibiotic resistance, antibiotics, gene sequencing, genomics, persisters, regulation of gene expression, tryptophan operon, viable but non-culturable cells

## Abstract

Persister and viable but non-culturable (VBNC) cells are two clonal subpopulations that can survive multidrug exposure via a plethora of putative molecular mechanisms. Here, we combine microfluidics, time-lapse microscopy, and a plasmid-encoded fluorescent pH reporter to measure the dynamics of the intracellular pH of individual persister, VBNC, and susceptible Escherichia coli cells in response to ampicillin treatment. We found that even before antibiotic exposure, persisters have a lower intracellular pH than those of VBNC and susceptible cells. We then investigated the molecular mechanisms underlying the observed differential pH regulation in persister E. coli cells and found that this is linked to the activity of the enzyme tryptophanase, which is encoded by *tnaA*. In fact, in a Δ*tnaA* strain, we found no difference in intracellular pH between persister, VBNC, and susceptible E. coli cells. Whole-genome transcriptomic analysis revealed that, besides downregulating tryptophan metabolism, the Δ*tnaA* strain downregulated key pH homeostasis pathways, including the response to pH, oxidation reduction, and several carboxylic acid catabolism processes, compared to levels of expression in the parental strain. Our study sheds light on pH homeostasis, proving that the regulation of intracellular pH is not homogeneous within a clonal population, with a subset of cells displaying a differential pH regulation to perform dedicated functions, including survival after antibiotic treatment.

## INTRODUCTION

Fluctuations in environmental conditions can result in phenotypic heterogeneity, for example, in terms of growth rate (1, 2), metabolic activity (3–5), or resistance to stress (6–9), within a clonal population. These cell-to-cell differences allow the population to withstand the challenges posed by dynamic shifts in the environment (10). For example, phenotypic heterogeneity plays an important role in population responses to antibiotic treatment (6, 11). Within an isogenic population, there are at least two phenotypic subpopulations of cells which are able to survive an antibiotic challenge, unlike in the majority of the susceptible population: persister and viable but non-culturable (VBNC) cells (12).

Persisters were first identified by Hobby et al. and Bigger almost 80 years ago, when penicillin was unable to completely sterilize bacterial cultures (13, 14), whereas VBNC cells were identified in 1982 by Xu and coworkers (15). These two subpopulations are able to survive antibiotic exposure. However, whereas persisters are able to resume growth once the antibiotic has been removed, VBNC cells do not resume growth when the antibiotic is removed but might regrow either after a long period of time (from days to months) or when specific conditions, for example, temperature and nutrients, are met (16–19). It is also worth noting that a closely related phenotype, a nongrowing-but-metabolically active state in Mycobacterium tuberculosis, is implicated in latent tuberculosis infections and relapses following chemotherapy (20). Two types of persisters have been identified in the literature: triggered and spontaneous, previously called type I and type II, respectively (6, 21). Spontaneous persisters arise during normal growth, unlike triggered persisters, which are generated by stress signals, such as starvation and antibiotic exposure (21). The eventual progenies of both persisters and VBNC cells are as susceptible to antibiotics as the original ancestral culture (14). This feature distinguishes these cells from genetically resistant mutants which possess genetic molecular elements that allow them to survive and replicate during antibiotic treatment (14, 22).

Persister and VBNC subpopulations pose a serious threat to human health and have been associated with chronic infections in immunocompromised patients affected by cystic fibrosis or HIV or those undergoing cancer chemotherapy (12, 23, 24). Persistence has also been linked to the relapse of recurrent diseases, including tuberculosis, candidiasis, salmonellosis, and the diseases caused by Chlamydia (25, 26), as well as infections of indwelling devices, such as catheters and heart valves (27). Moreover, there is increasing evidence of a profound link between these antibiotic-surviving phenotypes and the emergence of genetically resistant mutants (28–30).

The exact molecular mechanism for the generation of persister and VBNC cells remains unknown. It has been proposed that stochasticity in the expression of a variety of molecular pathways, such as toxin-antitoxin modules (31–33), the SOS response (34), the alarmone guanosine tetraphosphate (p)ppGpp pathway (33, 35–40), efflux pumps (41), and indole signaling (42–45), may play a role.

Unlike with the previously mentioned pathways, little is known about the role played by intracellular pH heterogeneity within a clonal population with regard to the survival of individual bacteria after an antibiotic challenge (46). The majority of aerobic bacteria, including Escherichia coli, are able to grow over a wide range of external pH values, from pH 5.5 to 9 (47, 48). However, excluding extremophiles, most bacteria maintain a near-neutral intracellular pH, this being a vital component of cellular physiology which bacteria need to tightly control due to the sensitivity of their intracellular enzymatic reactions (47, 49). In addition, metabolic reactions within the cytosol generate protons due to ATP production by glycolysis and oxidative phosphorylation. These processes cause an increase in acidity that the cell counteracts by expelling protons out of the cytosol via proton pumps and remodeling of key metabolic pathways (49–51). This helps maintain a stable electrochemical gradient across the cell membrane, which is vital for respiration (49). The cell regulates intracellular pH not only against internal pH variations but also in response to external stressors. For example, enteric bacteria, such as E. coli, must be able to survive in extreme pH ranges, as they need to pass through the stomach to reach a suitable environment for growth and infection (49). Shifting external pH values toward more extreme alkaline or acidic values induces the activation of *rpoS*, the SOS response, and heat shock-like responses, as well as cell-to-cell communication (52–55).

Specifically, cell-to-cell signaling via indole, a ubiquitous signaling molecule produced by over 85 species of bacteria, has recently been linked to the regulation of intracellular pH at the population level (56–58). Indole is synthesized by the enzyme tryptophanase (TnaA), which catalyzes the reversible conversion of tryptophan to indole, ammonia, and pyruvate (59). Indole signaling is involved in a variety of biological processes, such as the regulation of the pathogenicity of cells (60), the tightening of epithelial cell junctions (61), biofilm formation (59, 62), the activation of efflux pumps (44, 45), responses to virulence (44, 63), heat shock (101), and antibiotic stress (64). Two types of indole signaling have been identified: persistent indole signaling, when indole is present at a concentration of around 0.5 mM in the culture medium, and pulse indole signaling, occurring at the transition from the exponential to stationary phases with a maximum intracellular indole concentration of 50 mM, which can be mimicked experimentally by adding 5 mM indole to the culture medium (58). There is an ongoing debate regarding the role played by indole and tryptophan metabolism in antibiotic persistence, with some studies reporting that indole increases the fraction of persisters (65, 66) and others reporting the opposite (42, 43, 67). It is conceivable that these opposing findings might be due to the fact that inhibiting tryptophan metabolism (by genetically knocking out *tnaA* in E. coli) might have other profound consequences on bacterial metabolism and pH homeostasis, besides indole production; however, this hypothesis remains to be tested.

In this paper, we set out to quantify the heterogeneity in intracellular pH within a clonal E. coli population, aiming to establish whether there is a link between the heterogeneity in intracellular pH and antibiotic survival, which ultimately can dictate the phenotypic structure within a clonal population. Furthermore, we set out to investigate whether the intracellular bacterial pH could be manipulated by interfering with tryptophan metabolism and to determine the genetic and metabolic consequences of the lack of tryptophanase. In order to achieve these aims, we use a microfluidics-microscopy approach (8, 52) in combination with an intracellular pH reporter strain of E. coli to investigate the intracellular pH profiles of individual bacteria over time (8, 52). This approach allows us to determine the differences in intracellular pH before, during, and after ampicillin treatment in persister, VBNC, and susceptible bacteria (8, 52). Importantly, we considered bacteria to be VBNC if, after 3 h of antibiotic treatment and 21 h of exposure to antibiotic-free medium, they did not stain with propidium iodide, actively expressed fluorescent proteins, and did not divide within our experimental time frame. This does not exclude the possibility that these cells can start dividing if exposed to nutrients for longer periods of time (19, 68), whereas we can exclude the possibility that these cells are dead (69) since they actively express fluorescent proteins. Next, we used a tryptophanase knockout mutant strain in combination with whole-genome transcriptome analysis and exogenous indole supplementation to investigate the role played by tryptophan metabolism as well as indole signaling on phenotypic survival after exposure to ampicillin and on the maintenance of intracellular pH in the different phenotypic responses to ampicillin. Our data show that cell-to-cell differences in the regulation of intracellular pH represents one of the possible bacterial strategies for escaping antibiotic treatment and therefore might open the way to future therapeutics to eradicate persister bacteria. Furthermore, this novel approach opens the way for investigating the role of the differential regulation of intracellular pH in individual cells on dynamic responses of microbial populations to external stressors.

## RESULTS

### E. coli persisters display a lower intracellular pH than those of VBNC and susceptible cells before ampicillin treatment.

**FIG 1:**
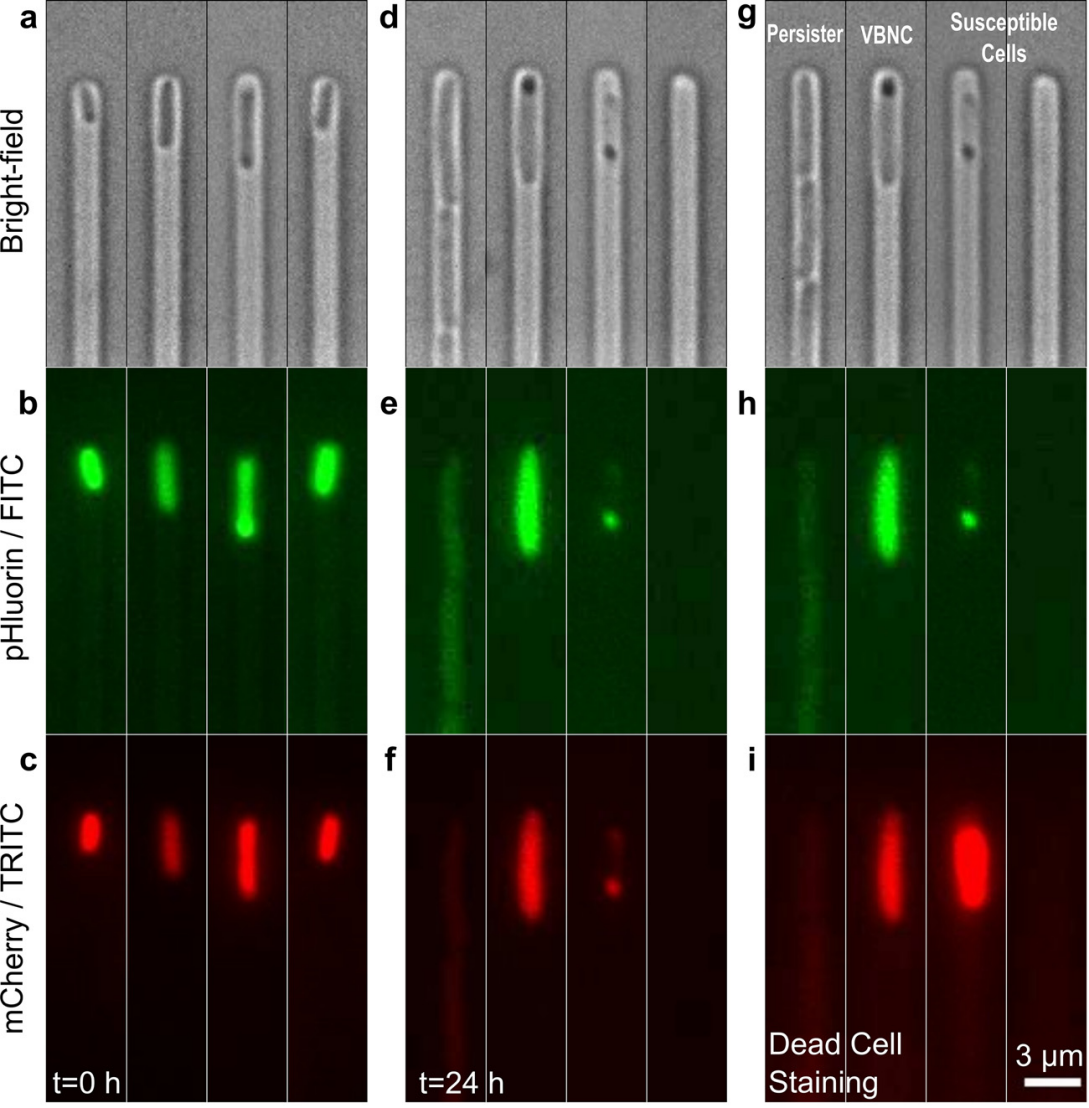
Fluorescence measurement of intracellular pH in individual persister, VBNC, or susceptible E. coli cells before and after ampicillin treatment. (a to c) Bright-field, FITC, and TRITC fluorescence images of four representative bacteria injected in the microfluidic mother machine from a 2 μl aliquot that was prepared as follows. A 200 ml 17 h stationary phase E. coli BW25113 culture, with a typical OD_595_ of 5, was spun down and resuspended in filtered medium (from the overnight culture) to be adjusted to an OD of 75. 2 μl were withdrawn from this sample and injected into the mother machine. (d to f) Corresponding images of the same bacteria after 3 h of incubation in ampicillin at a concentration of 25× the MIC in an M9-LB (90:10, vol/vol) solution and a successive 21 h of incubation with fresh nutrients. (g to i) Corresponding images of the same bacteria after a 20 min incubation in propidium iodide (PI) to distinguish between susceptible bacteria (second channel from the right), in which PI addition increases the mCherry red fluorescence, and VBNC bacteria (third channel from the right), in which PI addition has no effect on mCherry red fluorescence (Fig. S1). The dynamics of intracellular pH for individual cells in each phenotype was measured by removing the background fluorescence, normalizing the pHluorin against the mCherry signal for each cell at each time point, and using a pH calibration standard (see Materials and Methods). Scale bar, 3 μm.

Using time-lapse microscopy, we measured the temporal dynamics of the fluorescence levels of a pH-sensitive green fluorescent protein (GFP) (70, 71), pHluorin, for thousands of individual bacteria as a proxy for intracellular pH (see Materials and Methods). We also measured pHluorin-fused mCherry fluorescence to allow for normalization of any cell-to-cell heterogeneity in protein expression and plasmid copy number. It is worth noting that the fluorescent signals from pHluorin and mCherry were measured in series rather than in parallel by using two different filter sets that allowed for the excitation and the recording of the emission intensity of one of the two fluorophores at a time. This minimizes any artifacts due to potential fluorescence resonance energy transfer between the two fluorophores (72). These microscopy measurements were carried out using a microfluidic mother machine device (73) to image and track each individual bacterium before (time zero [*t *= 0 h]) (Fig. 1a to c) and during (0 < *t *< 3 h) exposure to ampicillin at a concentration of 25× the MIC, as well as during 21 h of exposure to lysogeny broth (LB) (3 < *t *< 24 h) (Fig. 1d to f and Materials and Methods). The mother machine device has previously been employed to investigate bacterial physiology and response to drugs (2, 9, 11, 73–75), and it has been demonstrated that this approach does not introduce cellular stress since both the doubling time and the fraction of persisters measured in the mother machine are in accordance with corresponding measurements performed on cultures growing in flasks or agar plates (9, 73). This single-cell approach allows us to determine the phenotype of each individual bacterium by flowing propidium iodide through the mother machine at 24 h, as previously reported. These phenotypes included persisters (leftmost channel in Fig. 1g), VBNC cells (second channel from the left in Fig. 1g), and susceptible bacteria (two rightmost channels in Fig. 1g) (9). The bacterium in the rightmost channel of Fig. 1g has lysed and disappeared from the channel after ampicillin treatment, whereas the bacterium in the second channel from the right was still visible in the channel but stained with propidium iodide and displayed significantly lower fluorescent protein expression than the VBNC cell; therefore, it was classed as susceptible (Fig. 1f and 1i, Materials and Methods; see also Fig. S1 in the supplemental material) (9). Moreover, the persister progeny were readily killed when exposed to ampicillin a second time at a concentration of 25× MIC at 24 h, confirming that persisters were phenotypically but not genetically resistant to antibiotics.

10.1128/mBio.00909-21.1 FIG S1 Mean red (i.e., TRITC filter) channel fluorescence of single-cell viable but non-culturable (VBNC) and susceptible-but-not-lysed bacteria before and after propidium iodide (PI) exposure. E. coli cells were exposed to 3 h of 25× the MIC of ampicillin before incubation for 21 h in fresh lysogeny medium. Images were captured at *t *= 24 h and then again after incubation in PI. VBNC cells display a significantly higher mCherry fluorescence than susceptible-but-not-lysed cells before incubation in PI. Susceptible-but-not-lysed bacteria have a significant increase in fluorescence after the addition of PI, indicating membrane damage. In contrast, the fluorescence of VBNC cells did not significantly change with the addition of PI. *n*_VBNC cells_ = 10, *n*_susceptible-but-not-lysed cells_ = 10. **, *P < *0.009; ****, *P < *0.0001. Download FIG S1, DOCX file, 0.1 MB. Copyright © 2021 Goode et al. 2021 Goode et al. https://creativecommons.org/licenses/by/4.0/ This content is distributed under the terms of the Creative Commons Attribution 4.0 International license.

**FIG 2:**
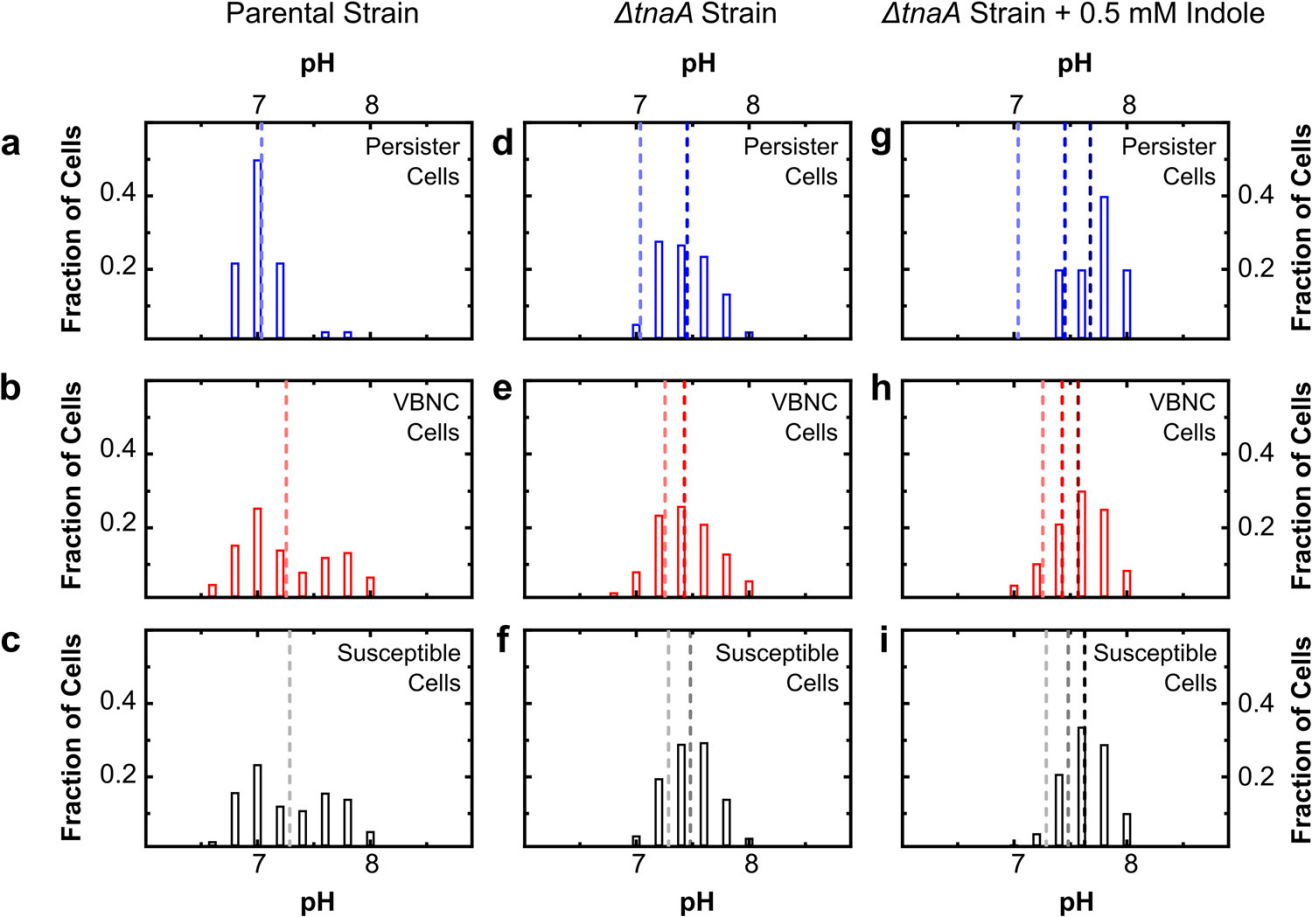
Before ampicillin treatment, persister E. coli cells exhibit a more acidic distribution of intracellular pH than susceptible and VBNC E. coli cells in an indole-positive strain. (a to c) Distributions of single-cell intracellular pH levels before drug treatment for persister (a), VBNC (b), and susceptible (c) bacteria of the parental E. coli strain. (d to f) Corresponding distributions of intracellular pH values for bacteria from the Δ*tnaA* E. coli strain. (g to i) Corresponding distributions of intracellular pH values for bacteria from the Δ*tnaA* E. coli strain with 0.5 mM indole supplementation (see Materials and Methods). Data are collated from at least three biological replicates, with the means of each data set depicted as dashed lines. Each replicate displayed a distribution of intracellular pH values similar to that displayed in the figure. Means of results from panels a to c and d to f are reproduced in panels g to i for comparison purposes only, showing that for all three phenotypes, the parental strain has the most acidic pH and the indole-supplemented Δ*tnaA* mutant has the most alkaline pH. Each strain was cultured at 37 °C for 17 h in LB before injection into a microfluidic device for single-cell pH analysis and subsequent phenotype determination using fluorescence microscopy, as illustrated in Fig. 1 and described in Materials and Methods. For the parental strain, *n*_persister cells_ was 30, *n*_VBNC cells_ was 104, and *n*_susceptible cells_ was 2,770. For the Δ*tnaA* strain, *n*_persister cells_ was 97, *n*_VBNC cells_ was 123, and *n*_susceptible cells_ was 2,907. For the Δ*tnaA* strain with indole, *n*_persister cells_ was 5, *n*_VBNC cells_ was 222, and *n*_susceptible cells_ was 4,729.

Crucially, at *t *= 0 h, before ampicillin exposure, VBNC and susceptible E. coli subpopulations exhibited a wide range of intracellular pH values, from pH 6.5 to pH 8 (the number of VBNC cells [*n*_VBNC cells_] = 104, *n*_susceptible cells_ = 2,770; 3 biological replicates), forming a bimodal distribution with peaks around 7.0 and 7.6 (Fig. 2b and c, respectively). In contrast, the persister subpopulation had a tighter regulation of intracellular pH, with a single peak at pH 7.0 (*n*_persister cells_ = 30 in 3 biological replicates) (Fig. 2a). This translated into a more acidic intracellular pH in persisters than in VBNC and susceptible bacteria, with mean values of 7.04 ± 0.04, 7.26 ± 0.03, and 7.28 ± 0.01, respectively, before antibiotic treatment. This differential pH regulation between different bacterial phenotypes suggests that the intracellular pH might play a role in survival to drug exposure and thus deserves further investigation.

### Abolishing tryptophanase activity eliminates the difference in intracellular pH between persister, VBNC, and susceptible E. coli cells before ampicillin treatment.

We then tested whether tryptophanase activity and indole production, which have previously been linked to persistence (42, 43, 65–67), play a role in the regulation of intracellular pH in E. coli by carrying out the set of experiments described above with a Δ*tnaA* knockout strain (56). In the Δ*tnaA* strain, the initial intracellular pH values of persister, susceptible, and VBNC cells before ampicillin treatment were significantly higher than the corresponding values measured for the parental E. coli strain (*P < *0.0001), in accordance with previously reported measurements at the population level (56). Crucially, for all three phenotypes of the Δ*tnaA* strain, the intracellular pH distribution displayed a single peak at around 7.5 (Fig. 2e and f); thus, abolishing tryptophanase activity and indole production eliminated the difference in intracellular pH between persister, VBNC, and susceptible cells before antibiotic treatment.

We next set out to determine whether supplementation with indole of a culture of the Δ*tnaA* strain would restore the persister parental-strain phenotype. We chose to perform chemical complementation with exogenous indole, instead of genetic complementation, in order to be able to contrast the effects between the loss of tryptophanase activity and the absence of indole signaling on the regulation of intracellular pH and on the phenotypic composition of the E. coli population. We mimicked persistent indole signaling by adding 0.5 mM extracellular indole when inoculating the Δ*tnaA* strain culture in LB or by progressively adding extracellular indole to the growing culture via a syringe pump to recapitulate the indole concentration produced by a growing parental-strain culture (56). We mimicked pulse indole signaling by adding 5 mM extracellular indole for 20 min directly before antibiotic treatment (see Materials and Methods) (56). Pulse indole signaling did not significantly affect the distribution of intracellular pH of the Δ*tnaA* strain, whereas both persistent indole signaling mimics returned similar intracellular pH distributions that were shifted toward alkaline values with respect to the Δ*tnaA* strain (Fig. S2). Therefore, we collated the experimental values gathered by using the former two approaches and found that the initial intracellular pH values of the persister, VBNC, and susceptible cells had alkalinized compared to that of the Δ*tnaA* mutant in the absence of indole (Fig. 2d and g [persister cells], Fig. 2e and h [VBNC cells], and Fig. 2f and i [susceptible cells]).

10.1128/mBio.00909-21.2 FIG S2 Distribution of single-cell intracellular pH of the Δ*tnaA* mutant E. coli strain with three different indole supplementation methods before ampicillin treatment (*t *= 0 h in Fig. 1) measured via microfluidics and fluorescence microscopy, as illustrated in Fig. 1 and in Materials and Methods. (a to c) Distributions of intracellular pH values obtained when the Δ*tnaA* mutant had been exposed to 0.5 mM indole during the 17 h overnight growth. (d to f) Distributions of intracellular pH values obtained when the 17 h overnight Δ*tnaA* mutant culture had been exposed to 5 mM indole for 20 minutes only. (g to i) Distributions of intracellular pH values obtained when the Δ*tnaA* mutant had been exposed to a time-dependent concentration of indole during the 17 h overnight growth (which corresponded to the measured extracellular concentration of indole in a growing parental strain culture [56]). Data are presented from at least three biological replicates, with the means depicted as dotted lines. Download FIG S2, DOCX file, 0.1 MB. Copyright © 2021 Goode et al. 2021 Goode et al. https://creativecommons.org/licenses/by/4.0/ This content is distributed under the terms of the Creative Commons Attribution 4.0 International license.

Taken together, these results suggest that indole supplementation does not restore the intracellular pH profiles observed for persister, VBNC, and susceptible cells in the parental strain. In fact, the addition of indole exacerbates the mean alkalinity of all three phenotypes.

### Genetic and metabolic consequences of the absence of tryptophanase.

**FIG 3:**
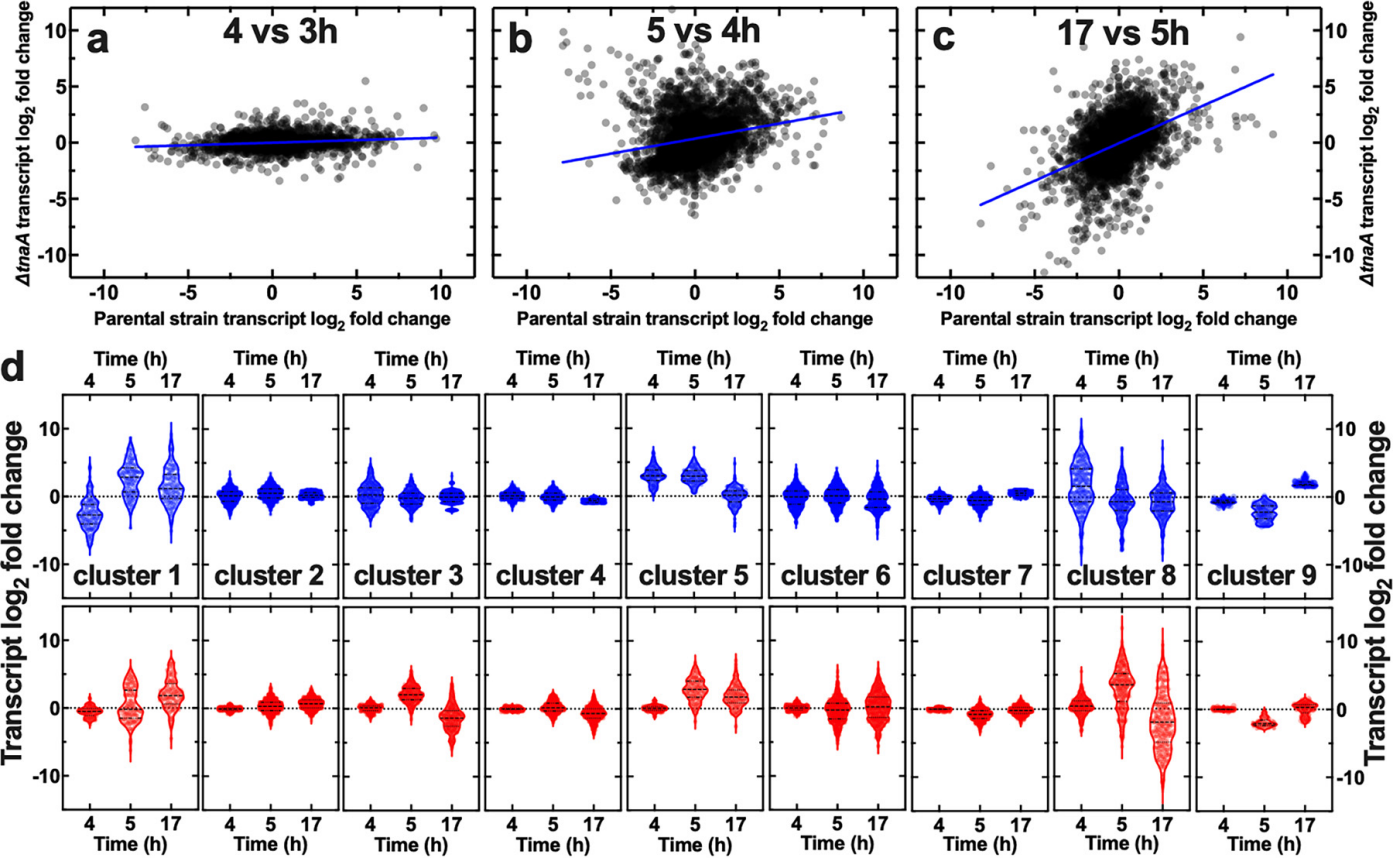
Genetic analysis of the consequences of the lack of tryptophanase. (a to c) Correlations between the log_2_ fold changes in transcript reads for the parental and Δ*tnaA* strains between 4 and 3 h (a), 5 and 4 h (b), and 17 and 5 h (c). Each dot is the log_2_ fold change in transcript reads for a single gene, and blue lines are linear regressions to the data returning Pearson correlation coefficient *R* values of 0.15, 0.23, and 0.43, respectively. Transcript reads were measured via RNA sequencing of samples in biological triplicates and are reported in Data Set S1. (d) Cluster analysis of the above-described transcriptomic data returned nine clusters with distinct patterns of gene regulation for the parental and Δ*tnaA* strains (top and bottom rows, respectively). Each dot represents the log_2_ fold change in transcript reads for a single gene, dashed lines indicate the median and quartiles of each distribution, and dotted lines indicate a log_2_ fold change of zero. The lists of genes belonging to each cluster are reported in Data Set S1.

After demonstrating that restoring extracellular indole alone does not restore the intracellular pH regulation observed in the parental strain, we set out to determine which other pathways are affected by the lack of tryptophanase besides the well-known conversion of tryptophan to indole, pyruvate, and ammonia. In order to determine the consequences of the loss of tryptophanase on gene regulation in E. coli, we performed genome-wide comparative transcriptome analysis between the parental and the Δ*tnaA* strain by following our previously reported protocols for RNA extraction, sequencing, and analysis from bacterial cultures grown in shaking flasks (see Materials and Methods and reference 76). Considering that *tnaA* expression is strongly upregulated at the transition from exponential to stationary phase (76), we performed RNA sequencing of biological triplicate samples after 3, 4, 5, and 17 h of growth in LB for both the parental and the Δ*tnaA* strain. At the whole-genome level, we found a weak correlation between the log_2_ fold change in gene expression in the parental and the Δ*tnaA* strains between 3 and 4 h (Fig. 3a) (*R* = 0.15), with the mutant displaying a significantly weaker regulation than the parental strain. Remarkably, this is the temporal window when *tnaA* is most strongly upregulated in the parental strain (6.1 ± 1.1 log_2_ fold change) (Data Set S1). In contrast, we found a stronger correlation between the log_2_ fold change in gene expression in the parental and the Δ*tnaA* strain between 4 and 5 h and between 5 and 17 h (Fig. 3b and c) (*R* = 0.23 and 0.44, respectively) when *tnaA* is downregulated in the parental strain (−0.7 ± 1.1 and −5.6 ± 1.1, respectively). These data suggest a major remodeling of gene regulation in E. coli due to the absence of tryptophanase in the 3 to 4 h temporal window, a point on which we expand below.

10.1128/mBio.00909-21.8 DATA SET S1 Gene regulation in the parental and Δ*tnaA* strains. Columns A and B report the list of genes and protein products, columns C and D report each gene base mean and cluster classification, and columns F to AB report the log_2_ fold changes, the standard errors for log_2_ fold changes (lfcSE), and adjusted *P* values (padj) at 4 versus 3 h, 5 versus 4 h, and 17 versus 5 h for the parental and Δ*tnaA* strains. Download Data Set S1, XLSX file, 1.1 MB. Copyright © 2021 Goode et al. 2021 Goode et al. https://creativecommons.org/licenses/by/4.0/ This content is distributed under the terms of the Creative Commons Attribution 4.0 International license.

**FIG 4:**
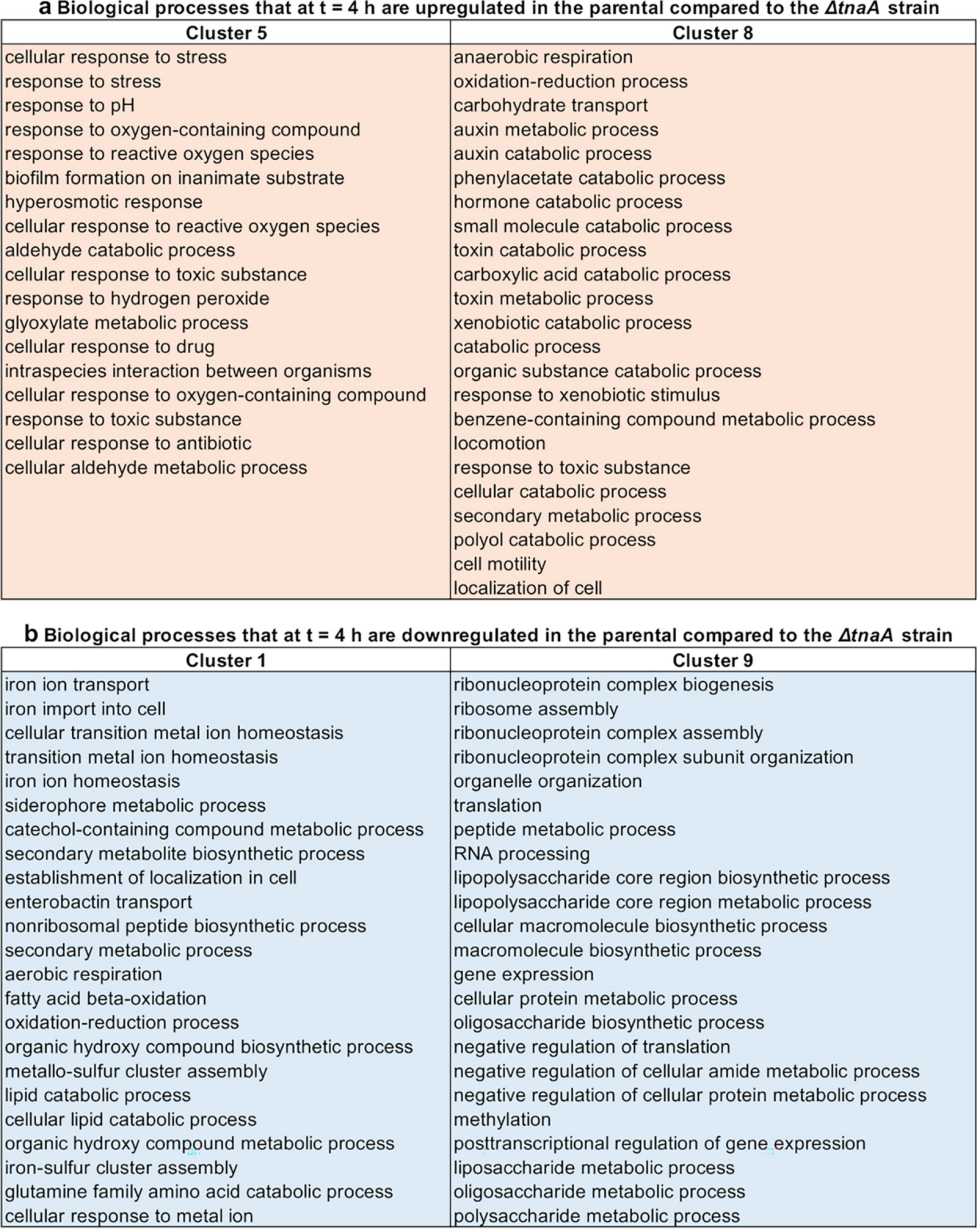
Biological processes differentially regulated as a consequence of the lack of tryptophanase. (a) Biological processes significantly overrepresented in gene clusters 5 and 8 (Fig. 3d), which contain genes that at *t* = 4 h are upregulated in the parental compared to the Δ*tnaA* strain. (b) Biological processes significantly overrepresented in gene clusters 1 and 9 (Fig. 3d), containing genes that at *t *= 4 h are downregulated in the parental compared to the Δ*tnaA* strain.

In order to identify biological processes that were differentially regulated between the Δ*tnaA* and the parental strain, we clustered the transcriptomic data above, identifying 9 distinct patterns of gene regulation for the parental and Δ*tnaA* strains (top and bottom rows in Fig. 3d, respectively, and Data Set S1). Clusters 5 and 8 most closely resembled *tnaA* regulation in the parental strain (Data Set S1), containing genes with a significantly stronger upregulation at *t *= 4 h in the parental than in the Δ*tnaA* strain (3.1 versus 0.1 and 1.7 versus 0.4 mean log_2_ fold changes, respectively; *P < *0.0001 for both) (Fig. 3d). Gene ontology analysis revealed that cluster 8 was enriched for anaerobic respiration, oxidation-reduction, carbohydrate transport, and several catabolic (including *tnaA*) processes (Fig. 4a). It is worth noting that at the transition from exponential to stationary phase, the environment around E. coli BW25113 becomes more acidic, with the extracellular pH reaching a minimum of 6.2 at *t *= 4 h, followed by alkalinization of the extracellular environment, with a maximum pH of 7.2 at *t *= 17 h (76). Furthermore, cluster 8 was enriched for carboxylic acid catabolic processes (e.g., *tnaA*, *ansB*, *garLR*, *pflB*, and *tdcB*) (Fig. 4a and Data Set S1) that at *t *= 4 h are upregulated in the parental but not in the Δ*tnaA* strain and that further lower the intracellular pH of the parental strain by producing organic acids, such as pyruvic acid (49).

In order to counteract this intracellular acidification, at *t *= 4 h, the parental but not the Δ*tnaA* strain upregulates oxidation-reduction and anaerobic respiration components, including cytochrome *bd* (*cydAB*), which contributes to the proton motive force, sulfate reductase (*cysH*), and formate (*fdnGHI*), glycerol (*gldA*), sorbitol (*srlD*), and NADH (*wrbA*) dehydrogenases, hydrogenases (*hyaA* and *hybABO*), and other proton-dependent catabolic processes, including lysine (*cadA*) and glutamate (*gadA*) decarboxylases (cluster 8 in Fig. 4a and Data Set S1). Several other proton-independent metabolic pathways are upregulated at *t *= 4 h in the parental but not in the Δ*tnaA* strain, including the fumarate (*frdBCD*), dimethyl sulfoxide (*dmsABC*), and nitrate and nitrite (*napA* and *narGHI*, *nirBD*) reductases and the propionate kinase (*tdcD*), as well as anion transport processes, such as alanine (*alaE*) and sulfate (*cysAPU*) transporters (cluster 8 in Data Set S1). Furthermore, gene ontology analysis revealed that cluster 5, which was also upregulated at *t *= 4 h in the parental but not in the Δ*tnaA* strain, was enriched in genes for the cellular response to stress, the response to pH, the response to reactive oxygen species, and hyperosmotic response processes (e.g., the aldehyde dehydrogenase *aldB*, the GABA permease *gabP*, the transcriptional regulator *gadX*, the glycine betaine transporter *osmF*, the superoxide dismutase *sodC*, the glutaminase *glsA*, the chaperone *hdeA*, and the deglycases *yhbO* and *hchA*) (cluster 5 in Fig. 4a and Data Set S1). Taken together, these data demonstrate that, thanks to the availability of tryptophanase, the parental strain is capable of performing key metabolic processes that allow the maintenance of a lower intracellular pH than the one displayed by the Δ*tnaA* strain, thus providing a mechanistic explanation of our data in Fig. 2 and previous bulk measurements (56).

**FIG 5:**
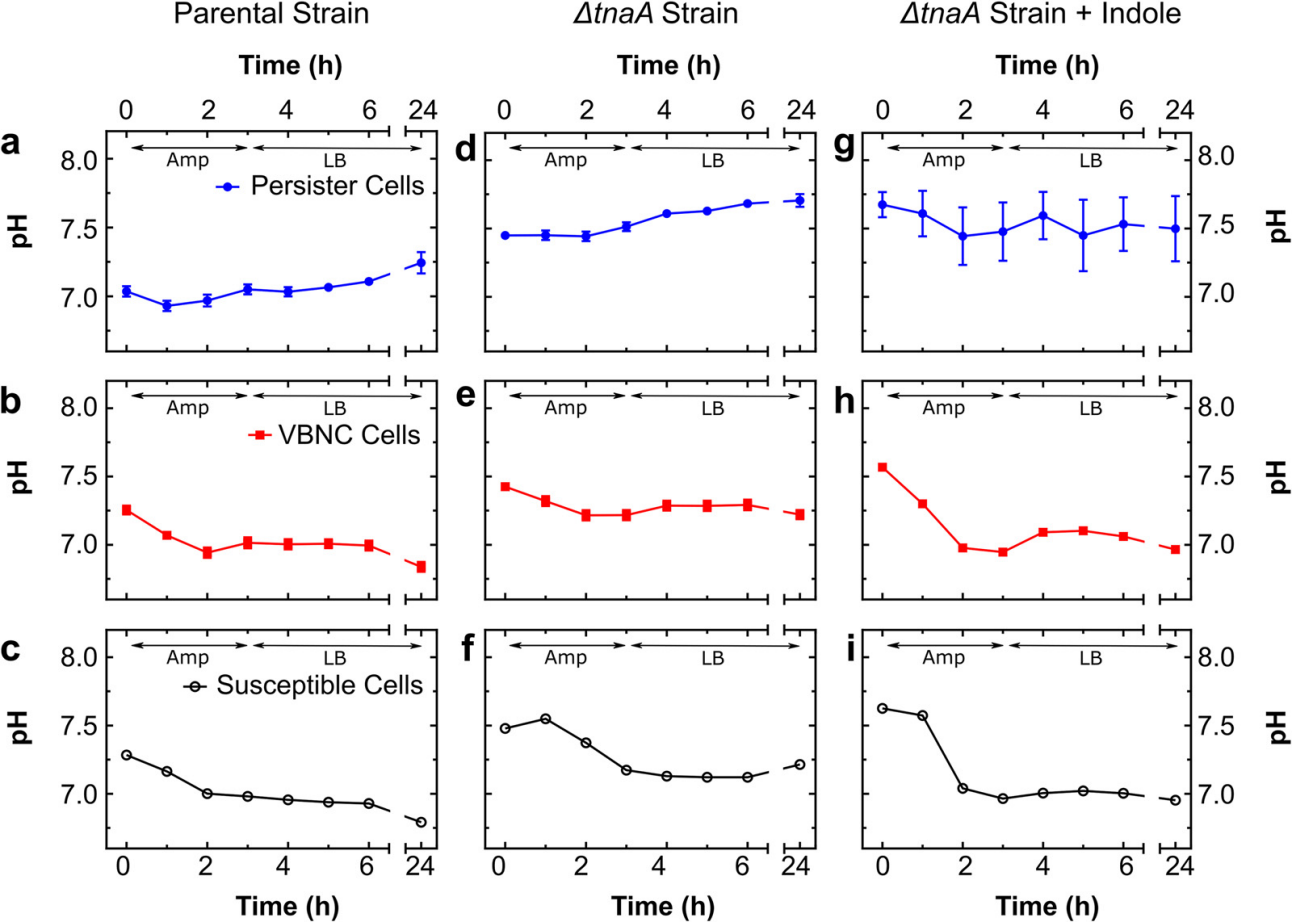
Ampicillin (Amp) treatment acidifies the intracellular pH of VBNC and susceptible bacteria but not of persisters. (a to c) Dynamics of intracellular pH of persister, VBNC, and susceptible E. coli cells before drug treatment (*t *= 0), during incubation in 25× the MIC of ampicillin in an M9-LB (90:10, vol/vol) solution (0 < *t *< 3 h), and during successive incubation in LB (3 < *t *< 24 h). (d to f) Corresponding dynamics of intracellular pH in a Δ*tnaA* E. coli strain. (g to i) Corresponding dynamics of intracellular pH in a Δ*tnaA* E. coli strain with 0.5 mM indole supplementation (see Materials and Methods). The data for each phenotype are presented as means and standard errors of the means from at least three biological replicates. Due to the large sample sizes (reported in Fig. 2), error bars are small compared to the corresponding mean values and are hidden behind some of the data points.

Clusters 1 and 9 displayed a significantly stronger downregulation at *t *= 4 h in the parental than in the Δ*tnaA* strain (−2.6 versus −0.6 and −0.7 versus 0.0 mean log_2_ fold changes, respectively; *P < *0.0001 for both) (Fig. 3d). Cluster 1 was primarily enriched in genes for metal ion transport and homeostasis (e.g., the *fec*, *fep*, and *fhu* operons, *tonB*, and *efeU*), secondary metabolism (e.g., the *ent* operon for the biosynthesis of the siderophore enterobactin), fatty acid metabolism (the fatty acid degradation regulon *fad*), and aerobic respiration (e.g., the cytochrome *bo* genes *cyoABCD*) processes, in contrast with cluster 8, which was enriched in genes for anaerobic respiration (Fig. 4b and Data Set S1). Cluster 9 was primarily enriched in genes for ribonucleoprotein and ribosome assembly (e.g., the 50S ribosomal protein L genes *rplABCDEFKP*), translation (e.g., the 30S ribosomal protein S genes *rpsDEGHKNPQS*), peptide metabolism (e.g., the elongation factor *tufA*), lipopolysaccharide metabolism (e.g., the lipopolysaccharide glucosyltransferase genes *waaBGJPQY*), and macromolecule and oligosaccharide biosynthesis processes (e.g., the transcription elongation factor *greA*) (Fig. 4b and Data Set S1). It is also worth noting that at *t *= 4 h, the Δ*tnaA* but not the parental strain upregulated carbohydrate transport processes in order to scavenge for carbon sources in the extracellular environment, especially the fructose-specific phosphotransferase system (*fru* operon) and, to a lesser extent, glucose (*ptsG*) and maltose (*mal* operon) import processes. Taken together, these data suggest that in order to compensate for the decreased availability of intracellular metabolites, such as pyruvate, which is a key intermediate in several metabolic pathways, at *t *= 4 h, the Δ*tnaA* strain employed alternative strategies (e.g., iron, carbohydrate, and peptide uptake and metabolism) for maintaining cellular metabolism and cell growth. Finally, clusters 2, 3, 4, 6, and 7 displayed a weak regulation at *t *= 4 h for both the parental and the Δ*tnaA* strain (Fig. 5d; Data Set S1), but it is worth noting that the mutant (but not the parental strain) used the cytochrome *bo* (*cyoABCD*) and proton antiporters (*chaA* and *mdfA*) to maintain pH homeostasis in the presence of the acidic extracellular pH.

Overall, this genome-wide transcriptome analysis revealed that abolishing tryptophanase activity causes a major remodeling of the E. coli gene expression profile besides abolishing the conversion of tryptophan to pyruvate, indole, and ammonia. Such remodeling includes key processes governing pH homeostasis, thus explaining our findings that the Δ*tnaA* strain displays a higher intracellular pH than the parental strain and that indole supplementation alone is not sufficient for lowering the intracellular pH of the mutant.

### Persister but not VBNC E. coli cells are capable of maintaining their intracellular pH during ampicillin treatment.

The average intracellular pH of VBNC and susceptible cells in the parental E. coli strain decreased as soon as 1 h after ampicillin addition at a concentration of 25× the MIC and reached a minimum of 7.0 after 3 h (Fig. 5b and c). Notably at *t *= 3 h, the second peak at pH 7.6 largely disappeared for susceptible and VBNC cells (Fig. S3). After this initial drop in intracellular pH, the VBNC and susceptible cells maintained an average intracellular pH at around 7.0 when incubated in fresh LB at *t *= 3 h, reaching a minimum pH of 6.8 at *t* = 24 h. In contrast, persisters maintained their intracellular pH during ampicillin treatment and then increased their pH during their successive growth on fresh nutrients, up to a maximum of 7.3 at *t* = 24 h (Fig. 5a and Fig. S3). Therefore, after treatment and incubation in LB, the distribution of intracellular pH within the E. coli population was completely reversed: persisters that before treatment had the most acidic mean pH were the most alkaline, and conversely, VBNC and susceptible E. coli cells that had the most alkaline pH were the most acidic.

10.1128/mBio.00909-21.3 FIG S3 Distributions of single-cell intracellular pH values before drug treatment for persister (a), VBNC (b), and susceptible (c) bacteria of the parental E. coli strain. (d to f) Corresponding distributions of intracellular pH values for bacteria after 3 h of ampicillin treatment at 25× the MIC. (g to i) Corresponding distributions of intracellular pH values for bacteria after 3 h ampicillin treatment and after 21 h of regrowth in fresh LB medium. Each strain was incubated at 37 °C for 17 h in lysogeny broth (LB) before injection into a microfluidic device for single-cell pH analysis and subsequent phenotype determination using fluorescence microscopy, as illustrated in Fig. 1 and described in Materials and Methods. The data presented in panel i represents susceptible nonlysed cells, as susceptible cells had lysed at this time point. Data are presented from at least three biological replicates, with the means depicted as dashed lines. Download FIG S3, DOCX file, 0.1 MB. Copyright © 2021 Goode et al. 2021 Goode et al. https://creativecommons.org/licenses/by/4.0/ This content is distributed under the terms of the Creative Commons Attribution 4.0 International license.

Similarly to the parental strain, the Δ*tnaA* mutant VBNC and susceptible cells exhibited a drop in intracellular pH after the addition of ampicillin (Fig. 5e and f). Both VBNC and susceptible cells exhibited two further phenotypic responses; a subset of VBNC and susceptible cells maintained a pH at around 7.6 at *t *= 3 h, whereas others exhibited a dramatically lower pH, at around 6.8 (Fig. S4). During the successive 21 h of incubation in fresh LB, VBNC and susceptible cells maintained an average intracellular pH of around 7.2 but still displayed two subpopulations at pHs 6.8 and 7.6 at *t *= 24 h (Fig. S4). The Δ*tnaA* persisters instead increased their pH after ampicillin addition, again as with the parental strain profile, up to a maximum of 7.7 at *t* = 24 h (Fig. 5d and Fig. S4).

10.1128/mBio.00909-21.4 FIG S4 Distributions of single-cell intracellular pH values before drug treatment for persister (a), VBNC (b), and susceptible (c) bacteria of the Δ*tnaA* mutant E. coli strain. (d to f) Corresponding distributions of intracellular pH values for bacteria after 3 h of ampicillin treatment at 25× the MIC. (g to i) Corresponding distributions of intracellular pH values for bacteria after 3 h ampicillin treatment and after 21 h of regrowth in fresh LB medium. Each strain was incubated at 37 °C for 17 h in lysogeny broth before injection into a microfluidic device for single-cell pH analysis and subsequent phenotype determination using fluorescence microscopy, as illustrated in Fig. 1 and described in Materials and Methods. Data are presented from at least three biological replicates, with the means depicted as dotted lines. Download FIG S4, DOCX file, 0.1 MB. Copyright © 2021 Goode et al. 2021 Goode et al. https://creativecommons.org/licenses/by/4.0/ This content is distributed under the terms of the Creative Commons Attribution 4.0 International license.

Finally, when we added 0.5 mM extracellular indole to the Δ*tnaA* mutant culture, we found a steep decrease of more than 0.5 in the intracellular pHs of VBNC and susceptible cells after ampicillin addition, down to a minimum of 7 (Fig. 5h and i). In contrast, we did not find a significant change in the intracellular pH of persisters throughout the experiment (Fig. 5g and Fig. S5).

10.1128/mBio.00909-21.5 FIG S5 Distributions of single-cell intracellular pH values before drug treatment for persister (a), VBNC (b), and susceptible (c) bacteria of the Δ*tnaA* E. coli mutant strain with extracellular indole supplementation (see Materials and Methods). (d to f) Corresponding distributions of intracellular pH values for bacteria after 3 h of ampicillin treatment at 25× the MIC. (g to i) Corresponding distributions of intracellular pH values for bacteria after 3 h ampicillin treatment and after 21 h of regrowth in fresh lysogeny broth. Each strain was cultured at 37 °C for 17 h in lysogeny broth before being injected into a microfluidic device for single-cell pH analysis and subsequent phenotype determination using fluorescence microscopy, as illustrated in Fig. 1 and described in Materials and Methods. Data are presented from at least three biological replicates, with the means depicted as dashed lines. Download FIG S5, DOCX file, 0.1 MB. Copyright © 2021 Goode et al. 2021 Goode et al. https://creativecommons.org/licenses/by/4.0/ This content is distributed under the terms of the Creative Commons Attribution 4.0 International license.

Taken together, these data demonstrate a striking difference between the intracellular pH regulation of persister cells and that of VBNC cells before, during, and after ampicillin treatment, with the VBNC cell intracellular pH profile resembling more closely that of the susceptible cells. Furthermore, these findings suggest that tryptophanase activity and the biological processes associated with it play a role in the regulation of intracellular pH before drug treatment but do not affect the dynamic profile of intracellular pH during antibiotic treatment, which is instead affected by supplementation of extracellular indole.

### Abolishing tryptophanase activity increases the fraction of persister but not VBNC E. coli cells.

**FIG 6:**
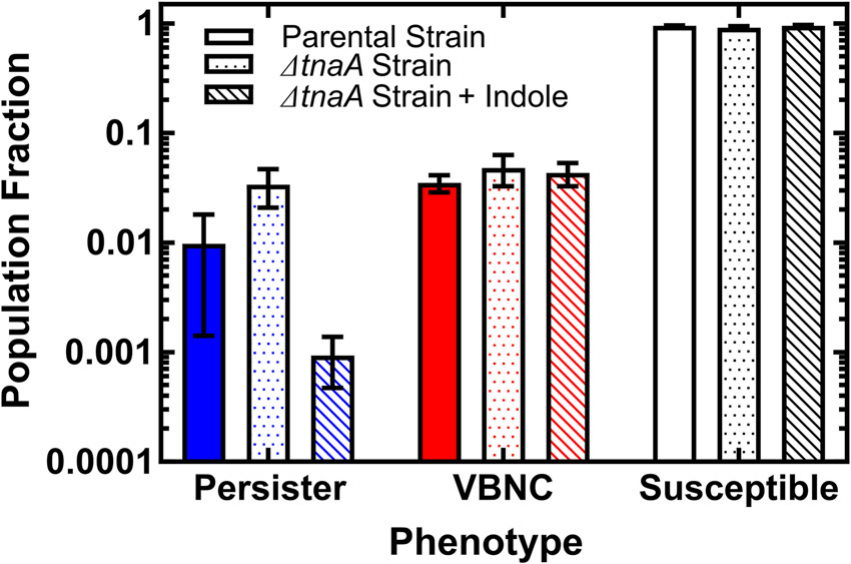
Extracellular indole reduces the fraction of persister but not of VBNC E. coli cells. Population fractions for persisters, VBNC cells, and susceptible parental strain E. coli cells (blue, red, and black filled bars, respectively) after a 3 h treatment with 25× the MIC of ampicillin. Corresponding population fractions measured for the Δ*tnaA* E. coli strain (dotted bars) and the Δ*tnaA* E. coli strain supplemented with 0.5 mM extracellular indole (hatched bars). Data are presented as means and standard errors of the means from at least 3 biological replicates.

Since our microfluidics-microscopy assay allows us to determine the fate of each individual cell after treatment with ampicillin at 25× the MIC, we were able to determine the composition of the microbial population in terms of the abundance of each phenotype in stationary phase, which is the phase investigated in all our single-cell measurements. We found that the fractions of persister, VBNC, and susceptible cells in the parental E. coli strain were 0.01, 0.04, and 0.95, respectively (filled bars in Fig. 6), in accordance with previously reported studies (9).

In comparison, the persister fraction of the Δ*tnaA* mutant was over three times higher than that of the parental strain, whereas the fraction of VBNC cells of the Δ*tnaA* mutant was similar to that measured for the parental strain (dotted and filled bars in Fig. 6, respectively). These data are in accordance with persister fractions found in bulk measurements showing that the Δ*tnaA* mutant contains more persisters than the parental strain in stationary phase, which is the growth phase after the indole pulse, which naturally occurs at the transition from exponential to stationary phase (Fig. S6a) (56, 57). In contrast, we found that the opposite occurred in bulk cultures in exponential phase, with a higher fraction of persisters in the parental strain than in the Δ*tnaA* mutant (Fig. S6b).

10.1128/mBio.00909-21.6 FIG S6 Percentages of survival of stationary phase (overnight culture diluted by spent medium to an OD_600_ of 0.15) (a) and exponential-phase (exponential culture at an OD_600_ of 0.15) (b) cells of parental (wild-type, indole-positive) and Δ*tnaA* (indole-negative) strains of E. coli BW25113 in LB medium treated for 5 h with 100× the MIC of ampicillin. Time zero samples were taken immediately before the antibiotic was added, and then cultures were incubated at 37 °C and 120 rpm and sampled after 1, 3, and 5 h. Samples were centrifuged for 7 min at 3,050 × *g*, and the cell pellet was washed twice with an equal volume of 1× PBS buffer to remove residual antibiotic. Washed samples were diluted in 1× PBS buffer (serial dilution), and 100 μl of two or more appropriate dilutions was spread on LB agar plates. The plates were incubated at 37 °C for 24 h, and the numbers of CFU were determined. The percentages of survival were calculated by comparing the numbers of CFU of samples after 1, 3, and 5 h of treatment to the number of CFU at time zero. The loss of indole production increased ampicillin persisters in stationary phase (after upregulation of tryptophan metabolism) but not in exponential phase (before upregulation of tryptophan metabolism). All data are means and standard deviations from a minimum of three biological replicates. Download FIG S6, DOCX file, 0.1 MB. Copyright © 2021 Goode et al. 2021 Goode et al. https://creativecommons.org/licenses/by/4.0/ This content is distributed under the terms of the Creative Commons Attribution 4.0 International license.

Next, we set out to determine whether supplementing a stationary phase Δ*tnaA* culture with 0.5 mM indole would restore the persister parental strain phenotype. When we supplemented the Δ*tnaA* mutant culture with indole, we found a decrease in the persister fraction, down to 0.001, which is more than an order of magnitude lower than that measured for the Δ*tnaA* strain in the absence of indole; in contrast, indole addition did not have an impact on the fraction of VBNC cells (Fig. 4). These data suggest that in the absence of tryptophanase, the number of stationary phase E. coli cells that are persisters after ampicillin treatment increases; however, chemically restoring persistent indole signaling restores the low persister fraction observed in the parental strain. Interestingly, we also note that neither tryptophanase activity nor indole signaling affected the composition of the microbial population in terms of the abundance of VBNC cells, which remained unchanged across the different experiments.

## DISCUSSION

Maintenance of intracellular pH within a cell is vital for cellular structure, functioning, and pathogenesis (49, 77). pH homeostasis allows correct protein folding, optimum enzymatic activity in metabolic reactions, and growth to take place (78), and it has recently been suggested that it might play a role in phenotypic survival after antibiotic treatments (46); however, this hypothesis remains to be tested. Therefore, we sought to investigate whether cell-to-cell differences in intracellular pH may represent a possible bacterial strategy to evade antibacterial activity.

By measuring the temporal changes in intracellular pH at the single-bacterium level, we found that before antibiotic exposure, E. coli persisters that survive ampicillin treatment display a lower intracellular pH than VBNC and susceptible E. coli cells. This difference resulted from a tighter regulation of pH in persisters, whereas VBNC and susceptible cells displayed a distribution of pH values, with two local maxima around 7.0 and 7.6. This novel finding represents, to the best of our knowledge, the first evidence of phenotypic differences between persister and VBNC cells before antibiotic treatment. Indeed, due to the difficulty of studying persisters and VBNC cells together, there is minimal literature comparing and contrasting these two phenotypes within the same experiment. The phenotypic differences that we found are notable considering that persister and VBNC cells are often thought to display the same dormant phenotype (26, 79), whereas we demonstrate here that persister and VBNC cells are clearly distinguishable, suggesting that antimicrobials that selectively target persisters could be developed. These data also shed new light on the idea proposed by Bartek et al. that a contributing factor of antibiotic lethality is an antibiotic’s effect on bacterial intracellular pH (77). Bartek et al. showed that a number of antibiotics cause an increase in the intracellular pH of Mycobacterium smegmatis, thought to disrupt essential processes, with the mechanism remaining unclear. This study also suggested that when a number of pathogens, including E. coli, were exposed to conditions which encouraged intracellular acidification, there was a decrease in their susceptibilities to antibiotics, including kanamycin, norfloxacin, and carbenicillin (77). This hypothesis proved to be true in our paper, at least with respect to persister E. coli cells displaying a lower pH than the population average before treatment and then surviving supra-MIC ampicillin treatment.

Persisters have been previously linked to indole and tryptophanase activities via a number of processes and stress responses, including multidrug exporters (44), dual-function importers (67), quorum sensing (60), oxidative shock (43, 65), and toxin-antitoxin modules (42). Therefore, we sought to determine the impact of the absence of tryptophanase activity and indole signaling on the intracellular pH values of persister, VBNC, and susceptible cells. Using a Δ*tnaA* strain, we found that the intracellular pH values of persister, susceptible, and VBNC cells before ampicillin treatment were significantly higher than the corresponding values measured for the parental E. coli strain, in accordance with population-level measurements (56). We then demonstrated that providing the Δ*tnaA* strain with mimics of persistent or pulse indole signaling could not restore the regulation of intracellular pH measured in the parental strain. Taken together, these data suggest that tryptophanase activity is key in maintaining pH homeostasis in response to changes in the pH of the extracellular environment (76) and that processes other than the conversion of tryptophan to indole underlie the observed acidification of stationary phase persisters, compared to VBNC or susceptible cells, preparing them to survive antibiotic challenge and promptly regrow after antibiotic removal.

Neutralophiles, such as E. coli, employ two major strategies to maintain pH homeostasis: the regulation of proton transporters, such as pumps and antiporters, and the remodeling of metabolic patterns, chiefly those involved in the production of organic acids (49). Accordingly, our whole-genome transcriptome and cluster analysis revealed that at the transition from exponential to stationary phase, the Δ*tnaA* strain upregulated a set of transporters that were orthogonal to those in the parental strain. Even more remarkably, the Δ*tnaA* strain significantly remodeled its metabolism compared to that of the parental strain, especially in terms of the use of amino acid decarboxylases, which are known to play a major role in acid resistance (80). Besides the loss of tryptophan conversion to pyruvic acid, which is known to decrease the intracellular pH (82), other key carboxylic acid catabolic processes, as well as oxidation-reduction, response to pH (83), response to reactive oxygen species, and hyperosmotic response components, were upregulated in the parental but not in the Δ*tnaA* strain. In contrast, the Δ*tnaA* but not the parental strain upregulated metal transport, secondary metabolism, fatty acid metabolism, ribosome assembly, translation, and carbohydrate transport processes. It is also worth noting that whereas the parental strain upregulated anaerobic respiration processes in this temporal window, the Δ*tnaA* strain, in contrast, upregulated aerobic respiration processes, and it is known that neutralophiles display different pH responses during anaerobic and aerobic respiration (83).

Overall, this novel knowledge about the genetic and metabolic consequences of the loss of tryptophanase is very important because it explains both our and previous findings that the Δ*tnaA* strain is more alkaline than the parental strain (56) and opens the way for the use of tryptophanase inhibitors (84) to manipulate bacterial metabolism and intracellular pH. Moreover, this novel genome-wide transcriptome analysis constitutes a very valuable resource when modeling bacterial metabolism in response to physicochemical changes in the extracellular environment (85–87), since we show that abolishing tryptophanase activity triggers a major remodeling of bacterial metabolism and pH homeostasis, whereas a previous study showed that indole supplementation alone affects protein expression to a lesser extent (88).

Finally, we sought to investigate the response to antibiotics in terms of both changes in intracellular pH in persister, VBNC, and susceptible cells as well as measuring the relative fractions of these three phenotypes after antibiotic treatment. We found that persisters increased their intracellular pH in the parental and Δ*tnaA* strains with and without indole supplementation, whereas VBNC and susceptible cells became more acidic in response to antibiotic treatment. Therefore, processes that are not linked to indole signaling or tryptophanase activity must underlie the alkalinization of persisters and acidification of VBNC cells in response to ampicillin treatment. It is worth noting that there are conflicting reports on the effect that indole has on the composition of a population in terms of the relative abundances of persister, VBNC, and susceptible cells. Vega et al. (65) and Kuczyńska-Wiśnik et al. (66) found an increase in the abundance of persisters in the presence of indole, whereas Hu et al. (42), Kwan et al. (43), and Wang et al. (67) found the opposite. These inconsistencies are probably due to the different indole concentrations, modes of action of antibiotics applied, types of indole signaling, and phases of cells used between assays (58). Indeed, we also observed a change in persister fraction within our ensemble measurements, depending on certain criteria. In exponential phase, we found a higher proportion of persisters in the parental than in the Δ*tnaA* strain. However, in stationary phase, this phenomenon was reversed, with a higher proportion of persisters found in the Δ*tnaA* than in the parental strain, but mimics of persistent indole signaling restored the persister levels measured for the parental strain. Therefore, the Δ*tnaA* strain appears to be a high-persister-level mutant (21), exhibiting high persister levels in stationary but not in exponential phase, suggesting that persistent indole signaling increases the level of triggered persisters but not of spontaneous persisters (89). Surprisingly, we found that the abundance of VBNC cells within the E. coli population was not affected by the removal of either tryptophanase activity or indole signaling. This is important because it contradicts the hypothesis that persister and VBNC cells display the same dormant phenotype (79).

In conclusion, our findings demonstrate that the regulation of intracellular pH is not homogeneous within a clonal population, with persisters displaying differential pH regulation and regrowing after antibiotic treatment. We showed that tryptophanase activity and several associated metabolic processes that are upregulated at the transition from exponential to stationary phase underpin this differential pH regulation, whereas tryptophanase activity and its associated indole signaling control the fraction of persister cells that survive and regrow after ampicillin treatment. Considering that more than 85 bacterial species encode tryptophanases (90), our data open the way for the manipulation of the intracellular pH and phenotypic compositions of microbial populations by developing and using tryptophanase-targeting compounds.

## MATERIALS AND METHODS

### Strains and culture conditions.

All chemicals were purchased from Fisher Scientific or Sigma-Aldrich unless otherwise stated. Cells were cultured using LB medium (10 g/liter tryptone, 5 g/liter yeast extract, and 10 g/liter NaCl; Melford) and LB agar plates (15 g/liter agar). Minimal medium (M9) was made from 1× M9 salts, 2 mM MgSO_4_, and 0.1 mM CaCl_2_ in deionized water. The medium was autoclaved before the addition of sterile thiamine hydrochloride, for a final concentration of 3 μM. E. coli BW25113 parental and Δ*tnaA* strains were obtained from the Keio collection (91, 92). Both strains were transformed with the pSCM002 plasmid, which expresses the mCherry-pHluorin translational fusion protein under an arabinose-inducible promoter and carries a chloramphenicol resistance marker. pSCM002 was constructed from pSCM001 (Addgene; 124605) (56) by swapping the ampicillin resistance marker with a chloramphenicol resistance marker. Briefly, PCR was used to amplify two fragments: a chloramphenicol resistance marker from pT2SC (Addgene 59382) and pSCM001, excluding the ampicillin resistance marker (93). Each fragment was amplified with flanking regions homologous to the other fragment. The two fragments were ligated using a Gibson assembly kit (NEB) (94) to generate a pBAD TOPO plasmid containing an mCherry-pHluorin translational fusion and a chloramphenicol resistance marker, pSCM002. The sequences of primers used to generate the pSCM002 plasmid are available in the supplemental material (Table S1). pHluorin (superecliptic pHluorin) is a green fluorescent reporter for intracellular pH derived from GFP with 8 amino acid changes (F64L, S65T, S147D, N149Q, T161I, S202F, Q204T, and A206T) (70, 71). mCherry fluorescence is not affected by pH and was therefore used to normalize changes in fluorescence caused by cell-to-cell variation in protein expression and differences in plasmid copy numbers between the exponential and stationary phases. Cultures were streaked on LB agar supplemented with chloramphenicol (25 μg/ml) for plasmid maintenance and incubated overnight at 37 °C to obtain single colonies. Overnight liquid cultures were prepared with a single colony from a streak plate and grown in 200 ml of LB medium supplemented with chloramphenicol (25 μg/ml) and arabinose (5 μg/ml) for 17 h at 37 °C on a shaking incubator at 200 rpm. When required, 0.5 mM indole (dissolved in absolute ethanol) was supplemented at the beginning of an overnight culture of the Δ*tnaA* strain.

10.1128/mBio.00909-21.7 TABLE S1 Primers used to generate the pSCM002 plasmid using Gibson assembly. Download Table S1, DOCX file, 0.01 MB. Copyright © 2021 Goode et al. 2021 Goode et al. https://creativecommons.org/licenses/by/4.0/ This content is distributed under the terms of the Creative Commons Attribution 4.0 International license.

### Microfluidic device fabrication.

The mother machine microfluidic device was fabricated by pouring a 9:1 (base-to-curing agent) polydimethylsiloxane (PDMS) mixture into an epoxy mold kindly provided by S. Jun, as previously described (95). The PDMS was cured at 70 °C for 2 h and then peeled from the epoxy mold to obtain 12 individual chips. Fluidic access was achieved using a 0.75 mm biopsy punch (Harris Uni-Core; WPI). The microfluidic device was assembled by bonding the PDMS chip to a glass coverslip, both of which were exposed to an air plasma treatment for 10 s at a 30 W plasma power (plasma etcher; Diener, Royal Oak, MI, USA) and then placed in contact to irreversibly bond the PDMS chip to the glass coverslip. The plasma treatment temporarily renders the device hydrophilic, so within 5 min postbonding, the device was filled with 2 μl of 50 mg/ml bovine serum albumin (BSA) solution and incubated at 37 °C for an hour to prevent cell adhesion to the internal surfaces of the device.

### Microfluidics-microscopy assay to measure intracellular pH in individual cells.

An overnight culture was prepared by incubating E. coli in LB at 37 °C for 17 h with agitation at 200 rpm. Bacteria were harvested from the overnight culture via centrifugation for 10 min at 4,000 rpm at room temperature (Eppendorf 5810 R). The supernatant was filtered twice (medical Millex-GS filter, 0.22 μm; Millipore Corp.) and used to resuspend the bacteria to an optical density at 595 nm (OD_595_) of 75; additionally, BSA was added to the bacterial suspension at a concentration of 0.5 mg/ml. Two microliters of the bacterial suspension was injected into the microfluidic device and incubated at 37 °C until there were 1 to 2 bacteria in the lateral side channels. Fluorinated ethylene propylene tubing (1/32 in. by 0.008 in.) was connected to the inlet and outlet holes and connected to a flow rate measuring device (Flow Unit S; Fluigent, Paris, France) and outlet reservoir, respectively. The flow rate measuring device was regulated by a computerized pressure-based flow control system (MFCS-4C; Fluigent) controlled by MAESFLO software (Fluigent). Spent medium was flushed through the device to wash excess bacteria out of the main channel at 300 μl/h for 8 min to completely exchange the fluid in the device and tubing. The chip was mounted on an inverted microscope (IX73 Olympus, Tokyo, Japan), and images were acquired in bright-field and two fluorescence modes. Images were collected via a 60×, 1.2 numerical aperture (NA) objective (UPLSAPO60XW; Olympus) and an sCMOS camera (Zyla 4.2; Andor, Belfast, UK). The fluorescence modes used a fluorescein isothiocyanate (FITC) and tetramethyl rhodamine isothiocyanate (TRITC) filter, exposing the bacteria to blue and green excitation bands of broad-spectrum LEDs at 20% and 5% of their intensity, respectively, for 0.03 s. The microfluidic device was moved by two automated stages (M-545.USC and P-545.3C7 [Physik Instrumente, Karlsruhe, Germany] for coarse and fine movements, respectively) to image multiple fields of view in a sequential manner. The imaging setup was controlled by LabView, with automated shutter and filter switching. After acquiring the first set of images, we allowed 25× the MIC of ampicillin to flow in a 90:10, vol/vol, M9-LB solution at 300 μl/h for 8 min before lowering the flow rate to 100 μl/h for 3 h. After 3 h of treatment, LB medium was flushed through the chip at 300 μl/h for 8 min before the rate was lowered to 50 μl/h for 21 h. Images were taken hourly between *t* = 0 and *t* = 6 h and at 24 h. At 24 h, propidium iodide (PI) was used to flow through the microfluidic device for 15 min according to the manufacturer’s specifications. Bright-field and fluorescence images were acquired to distinguish between VBNC and SNL cells using 5% green LED intensity with a TRITC filter for 0.03 s. All experiments were carried out at room temperature.

### Indole supplementation.

Three indole supplementation protocols were tested to recover the parental strain in the Δ*tnaA* strain. The first protocol used addition of indole to a final concentration of 0.5 mM at the start of a culture and then incubation overnight as previously described. The second protocol mimicked the previously specified indole pulse (56, 57) by supplementing indole at a final concentration of 5 mM for 20 min once the 17 h overnight Δ*tnaA* culture was loaded in the microfluidic device. The third protocol used a portable syringe pump (Chemyx Inc.) to dynamically drip indole into a growing Δ*tnaA* culture for 200 min to mimic the external indole concentration previously recorded by Zarkan et al. (56). The results from all three indole supplementation methodologies are reported in Fig. S2.

### Intracellular pH determination via single-cell fluorescence.

pHluorin fluorescence intensity has a linear relationship with pH between pH 6.5 and pH 8 (71). The fluorescence intensity of mCherry is unaffected by pH and was used to normalize the pHluorin fluorescence due to variations in protein expression and plasmid copy number between individual cells. To equate the relative fluorescence of each cell to a pH value, the intensity ratio of pHluorin to mCherry for each cell was calculated and, with a calibration curve, converted to a pH value. The standard curve was generated by exposing the parental strain and Δ*tnaA* mutant to 50 μM carbonyl cyanide *m*-chlorophenyl hydrazine (CCCP) at 300 μl/h for 8 min and then at 100 μl/h for 20 min, which causes the intracellular pH to equalize to the pH of the medium by allowing the translocation of protons across the cytoplasmic membrane (56). PBS solutions whose pHs were manually adjusted to 6.5, 7.0, 7.5, and 8.0 were used to generate the standard curve using the same imaging/experimental settings described above. The relationship between fluorescence and pH obtained from the standard curve was used to calibrate all pH values in our experiments.

### Image and data analysis.

All image analysis was performed using a custom Python module, MMHelper (96). In brief, channels and bacteria were detected by using automated thresholding algorithms and were assigned unique numeric labels. Each bacterium in a frame was tracked, and a lineage was established and then manually verified. Note the increase in red fluorescence of the susceptible but not of the VBNC cells, thus indicating the susceptibility of the cell. By normalizing the pH-sensitive pHluorin intensity against the mCherry intensity, which is unaffected by pH, for each bacterium at each time point, we accounted for variation in protein expression and plasmid copy number between individual cells. We determined the intracellular pH of individual cells from their relative fluorescence by using a calibration curve.

For each lineage, the corresponding masks were used to extract the width, length, area, and fluorescence intensities of the individual bacteria from the corresponding fluorescence images. The background fluorescence was established from the average fluorescence for the areas of the channels that did not contain bacteria and subtracted from the fluorescence intensity measured on each bacterium. The information for all bacteria within a frame across the different time points was written into .csv files. Each bacterium was labeled manually as a persister, a VBNC cell, a susceptible lysed cell, or a susceptible nonlysed cell. The data presented in this paper were plotted using GraphPad Prism 8 and represent means and standard errors of the means from at least 3 biological replicates. Due to the large sample sizes, error bars are small compared to the corresponding mean values and are hidden behind the data points in some of the graphs. Statistical significance was tested by an unpaired *t* test with Welch’s correction.

### Transcriptomic analysis.

RNA isolation, library preparation, sequencing, and transcriptomic data processing were performed as previously reported (76). Briefly, parental and Δ*tnaA* E. coli cultures were prepared as described above, and 500 μl aliquots were taken after *t *= 3, 4, 5, and 17 h after inoculation in LB in biological triplicates. RNAprotect bacterial reagent (Qiagen) was added to each aliquot. Extractions were performed using an RNeasy minikit (Qiagen) by following the manufacturer’s instructions. DNA removal during extraction was carried out by using RNase-Free DNase I (Qiagen). RNA concentration and quality were measured using a Qubit 1.0 fluorometer (ThermoFisher Scientific) and 2200 TapeStation (Agilent), respectively, and only samples with an RNA integrity number larger than 8 were taken forward. Transcript abundance was quantified using Salmon for each gene in all samples. Subsequent differential analysis was performed using DEseq2 in R software to quantify the log_2_ fold change in transcript reads (97) for each gene.

### Cluster and gene ontology analysis.

Clustering analysis of the transcriptome data was performed using the mclust 5 package (version 5.4.7) for R (98). Using this method, a Gaussian finite mixture model was estimated for all transcript log_2_ fold change data, with 6 total dimensions (3 time point comparisons across 2 genotypes). The method was configured to test all possible models (e.g., hyperspherical versus ellipsoidal cluster shapes, equal volume versus various volume clusters, etc.), for a range of 2 to 20 total clusters. The minimal, best-fitting model was identified by the Bayes information criterion as 9 ellipsoidal clusters with equal shapes and orientations. Gene Ontology enrichment analysis was performed using the clusterProfiler package (version 3.16.1) for R (99). Enrichment in terms belonging to the “Biological Process” ontology was calculated for each gene cluster, relative to the set of all genes quantified in the experiment, via a one-sided Fisher exact test (hypergeometric test). *P* values were adjusted for false discovery by using the method of Benjamini and Hochberg (100). Finally, the lists of significantly enriched terms were simplified to remove redundant terms, as assessed via their semantic similarity to other enriched terms, using clusterProfiler’s simplify function.
