# Supplementary material for: Persister Escherichia coli Cells Have a Lower Intracellular pH than Susceptible Cells but Maintain Their pH in Response to Antibiotic Treatment
Source: mBio. 2021 Jul 20;12(4):e00909-21. doi: 10.1128/mBio.00909-21 (PMC8406257; doi:10.1128/mBio.00909-21)
Supplement: FIG S1 [file mbio.00909-21-sf001.docx]

# Persister *E. coli* have a lower intracellular pH than susceptible cells but maintain their pH in response to antibiotic treatment

**Olivia Goode,^a,b^ Ashley Smith,^a,b^ Ashraf Zarkan,^c^  Jehangir Cama,^a,d^ Brandon M. Invergo,^e^ Daaniyah Belgami,^c^ Santiago Caño-Muñiz,^c,f^ Jeremy Metz,^a,b^ Paul O’Neill,^b^ Aaron Jeffries,^b^ Isobel H Norville,^g^ Jonathan David,^g^ David Summers,^c^ Stefano Pagliara^a,b^#**

^a^ Living Systems Institute, University of Exeter, Stocker Road, Exeter, EX4 4QD,

United Kingdom.

^b^ School of Biosciences, College of Life and Environmental Sciences, University of Exeter, Stocker Road, Exeter, EX4 4QD, United Kingdom.

^c^ Department of Genetics, University of Cambridge, Cambridge, CB2 3EH, United Kingdom.

^d^ College of Engineering, Mathematics and Physical Sciences, University of Exeter, Stocker Road, Exeter, EX4 4QD, United Kingdom.

^e^ Translational Research Exchange @ Exeter, University of Exeter, Stocker Road, Exeter, EX4 4QJ, United Kingdom.

^f^ MRC Laboratory of Molecular Biology, Cambridge, CB2 0QH, United Kingdom.

^g^ Dstl, Porton Down, Salisbury, SP4 0JQ, United Kingdom.

#Address correspondence to Stefano Pagliara, [s.pagliara@exeter.ac.uk](mailto:s.pagliara@exeter.ac.uk)


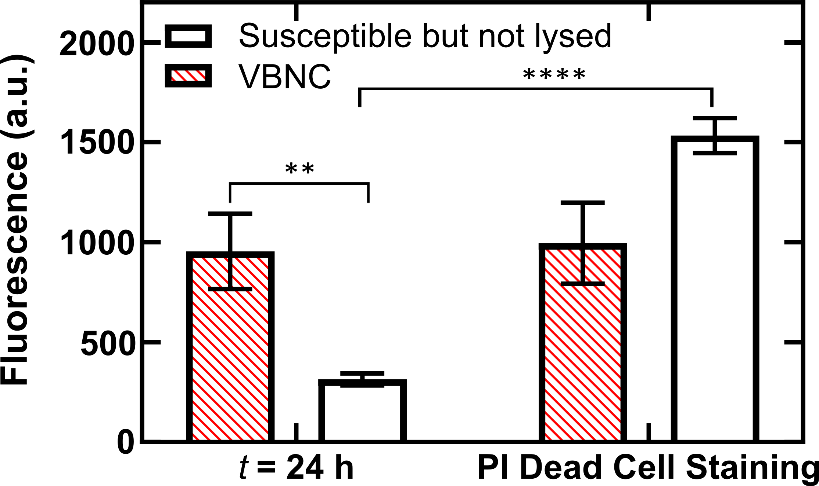


**Supplementary Figure 1.**

Mean red (i.e. TRITC filter) channel fluorescence of single-cell viable but non-culturable (VBNC) and susceptible but not lysed bacteria before and after propidium iodide (PI) exposure. *E. coli* cells were exposed to three hours of 25 × MIC ampicillin treatment before incubation for 21 h in fresh lysogeny medium. Images were captured at *t*=24 h and then again after incubation in propidium iodide (PI). VBNC cells display a significantly higher mCheerry fluorescence compared to susceptible but not lysed cells before incubation in PI. Susceptible but not lysed bacteria have a significant increase in fluorescence after the addition of PI indicating membrane damage. In contrast, the fluorescence of VBNC cells did not significantly change with the addition of PI. *N_VBNC_*=10, *n_susceptible but not lysed_*=10. **: *p*<0.009; ****: *p*<0.0001.
