# Supplementary material for: Persister Escherichia coli Cells Have a Lower Intracellular pH than Susceptible Cells but Maintain Their pH in Response to Antibiotic Treatment
Source: mBio. 2021 Jul 20;12(4):e00909-21. doi: 10.1128/mBio.00909-21 (PMC8406257; doi:10.1128/mBio.00909-21)
Supplement: FIG S3 [file mbio.00909-21-sf003.docx]

# Persister *E. coli* have a lower intracellular pH than susceptible cells but maintain their pH in response to antibiotic treatment

**Olivia Goode,^a,b^ Ashley Smith,^a,b^ Ashraf Zarkan,^c^  Jehangir Cama,^a,d^ Brandon M. Invergo,^e^ Daaniyah Belgami,^c^ Santiago Caño-Muñiz,^c,f^ Jeremy Metz,^a,b^ Paul O’Neill,^b^ Aaron Jeffries,^b^ Isobel H Norville,^g^ Jonathan David,^g^ David Summers,^c^ Stefano Pagliara^a,b^#**

^a^ Living Systems Institute, University of Exeter, Stocker Road, Exeter, EX4 4QD,

United Kingdom.

^b^ School of Biosciences, College of Life and Environmental Sciences, University of Exeter, Stocker Road, Exeter, EX4 4QD, United Kingdom.

^c^ Department of Genetics, University of Cambridge, Cambridge, CB2 3EH, United Kingdom.

^d^ College of Engineering, Mathematics and Physical Sciences, University of Exeter, Stocker Road, Exeter, EX4 4QD, United Kingdom.

^e^ Translational Research Exchange @ Exeter, University of Exeter, Stocker Road, Exeter, EX4 4QJ, United Kingdom.

^f^ MRC Laboratory of Molecular Biology, Cambridge, CB2 0QH, United Kingdom.

^g^ Dstl, Porton Down, Salisbury, SP4 0JQ, United Kingdom.

#Address correspondence to Stefano Pagliara, [s.pagliara@exeter.ac.uk](mailto:s.pagliara@exeter.ac.uk)


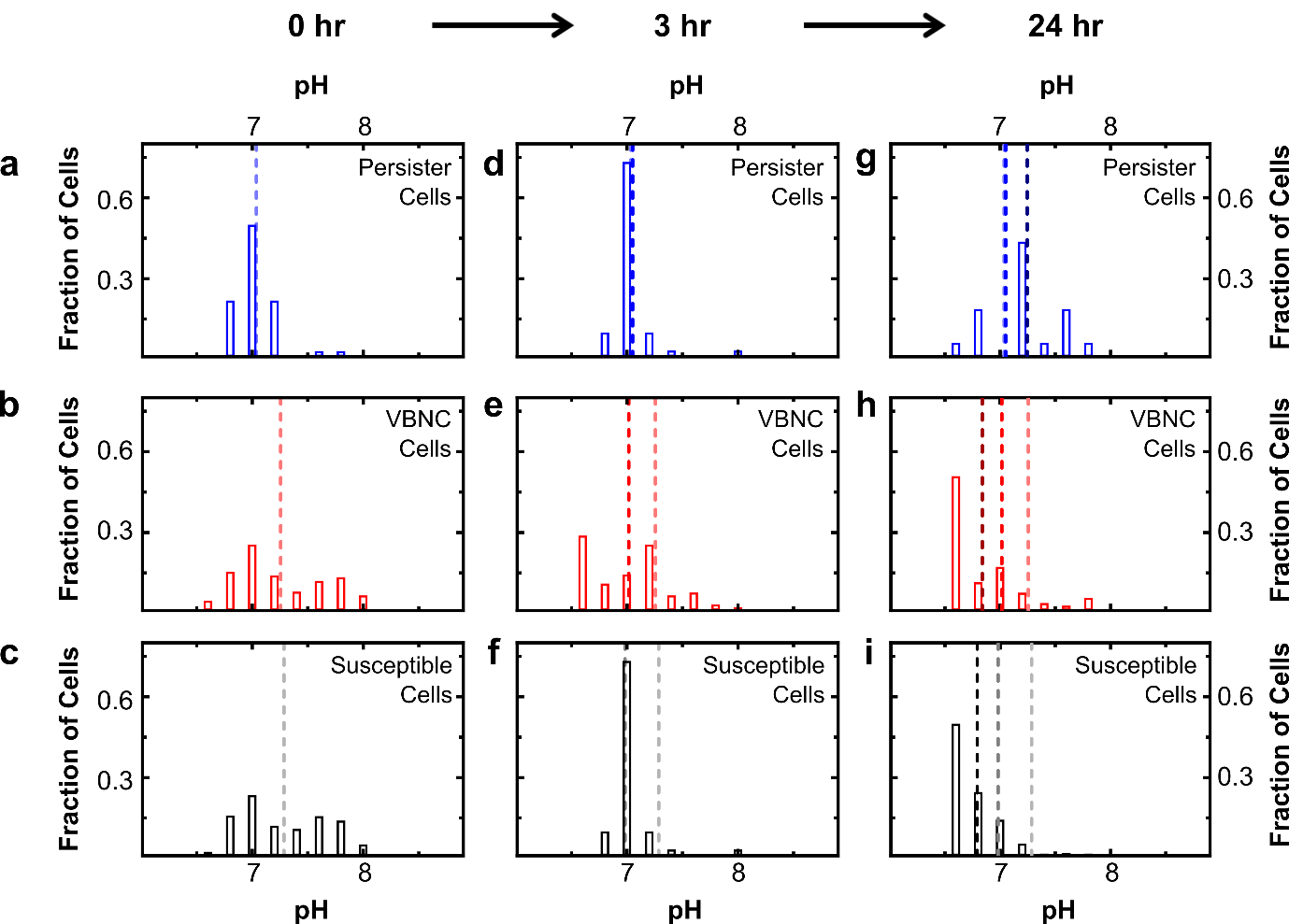


**Supplementary Figure 3.**

Distributions of single cell intracellular pH before drug treatment for **(a)** persister, **(b)** viable but non-culturable **(**VBNC) and **(c)** susceptible bacteria of the parental *E. coli* strain. **(d-f)** Corresponding distributions of intracellular pH values for bacteria after three hours of 25 × MIC ampicillin treatment. **(g-i)** Corresponding distributions of intracellular pH values for bacteria three hours after ampicillin treatment and 21 hours of regrowth in fresh LB media. Each strain was incubated at 37 °C for 17 hours in Lysogeny Broth (LB) before injection into a microfluidic device for single-cell pH analysis and subsequent phenotype determination using fluorescence microscopy as illustrated in **Fig. 1** and in the **Methods**. The data presented in panel **i** represents susceptible non-lysed cells as susceptible cells had lysed at this time point. Data is presented from at least three biological replicates with the means depicted as dotted lines.
