# Supplementary material for: Persister Escherichia coli Cells Have a Lower Intracellular pH than Susceptible Cells but Maintain Their pH in Response to Antibiotic Treatment
Source: mBio. 2021 Jul 20;12(4):e00909-21. doi: 10.1128/mBio.00909-21 (PMC8406257; doi:10.1128/mBio.00909-21)
Supplement: FIG S6 [file mbio.00909-21-sf006.docx]

# Persister *E. coli* have a lower intracellular pH than susceptible cells but maintain their pH in response to antibiotic treatment

**Olivia Goode,^a,b^ Ashley Smith,^a,b^ Ashraf Zarkan,^c^  Jehangir Cama,^a,d^ Brandon M. Invergo,^e^ Daaniyah Belgami,^c^ Santiago Caño-Muñiz,^c,f^ Jeremy Metz,^a,b^ Paul O’Neill,^b^ Aaron Jeffries,^b^ Isobel H Norville,^g^ Jonathan David,^g^ David Summers,^c^ Stefano Pagliara^a,b^#**

^a^ Living Systems Institute, University of Exeter, Stocker Road, Exeter, EX4 4QD,

United Kingdom.

^b^ School of Biosciences, College of Life and Environmental Sciences, University of Exeter, Stocker Road, Exeter, EX4 4QD, United Kingdom.

^c^ Department of Genetics, University of Cambridge, Cambridge, CB2 3EH, United Kingdom.

^d^ College of Engineering, Mathematics and Physical Sciences, University of Exeter, Stocker Road, Exeter, EX4 4QD, United Kingdom.

^e^ Translational Research Exchange @ Exeter, University of Exeter, Stocker Road, Exeter, EX4 4QJ, United Kingdom.

^f^ MRC Laboratory of Molecular Biology, Cambridge, CB2 0QH, United Kingdom.

^g^ Dstl, Porton Down, Salisbury, SP4 0JQ, United Kingdom.

#Address correspondence to Stefano Pagliara, [s.pagliara@exeter.ac.uk](mailto:s.pagliara@exeter.ac.uk)


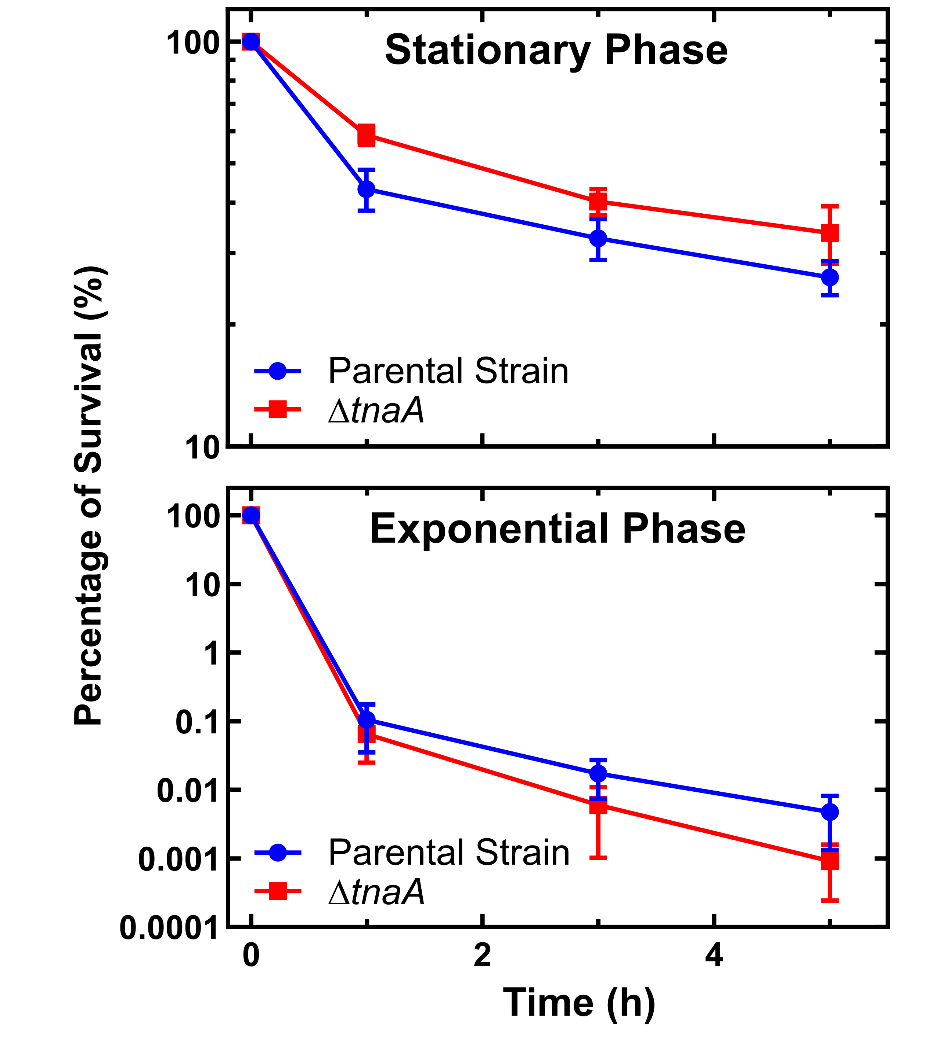


**Supplementary Figure 6.**

Percentage of survival of stationary phase (**a**) (overnight culture diluted by spent medium to OD600 = 0.15) and exponential phase (**b**) (exponential culture at OD600 = 0.15) of parental (wild type - indole positive) and *ΔtnaA* (indole negative) strains of BW25113 *E. coli* in LB medium treated for five hours with 100 × MIC ampicillin. Time zero samples were taken immediately before the antibiotic was added, and then cultures were incubated at 37°C, 120 rpm and sampled after 1, 3 and 5 hours. Samples were centrifuged for 7 min at 3,050 × g and the cell pellet was washed twice with an equal volume of 1 × PBS buffer to remove residual antibiotic. Washed samples were diluted in 1 × PBS buffer (serial dilution) and 100 μl of two or more appropriate dilutions were spread on LB agar plates. The plates were incubated at 37°C for 24 h and the CFU (colony forming units) were determined. The percentage of survival was calculated by comparing the CFU of samples after 1, 3 and 5 hours of treatment to the CFU at time zero. The loss of indole production increased ampicillin persisters in stationary phase (after up-regulation of tryptophan metabolism) but not in exponential phase (before up-regulation of tryptophan metabolism). All data are the means ± SD of a minimum of three biological replicates.
